# Glucagon-like Peptide-1 Receptor Agonists in Liver Transplant Recipients: A Retrospective Cohort Study

**DOI:** 10.1097/TXD.0000000000001932

**Published:** 2026-03-23

**Authors:** Hesham Sheashaa, Ramzi Ibrahim, Amani Elshaer, Hoang Nhat Pham, Rama Mouhaffel, Eiad Habib, Mahmoud Abdelnabi, Juan M. Farina, Steven J. Lester, David Simper, Said Alsidawi, Eric D. Steidley, Bashar A. Aqel, Michele Barnhill, W. Ray Kim, Chadi Ayoub, Reza Arsanjani

**Affiliations:** 1 Department of Cardiovascular Medicine, Mayo Clinic, Phoenix, AZ.; 2 Division of Gastroenterology and Hepatology, Mayo Clinic, Phoenix, AZ.; 3 Department of Cardiovascular Medicine, Mayo Clinic, Rochester, Minnesota, MN.; 4 Department of Medicine, University of Arizona, Tucson, AZ.; 5 Department of Medicine, Transplant Center, Mayo Clinic, Phoenix, AZ.

## Abstract

**Background.:**

Liver transplant recipients face high risks of cardiometabolic events after transplant, driven by posttransplant weight gain, diabetes, hypertension, as well as immunosuppression-related side effects. Glucagon-like peptide-1 receptor agonists (GLP1RAs) improve metabolic and cardiorenal outcomes in nontransplant populations, but their role in liver transplant recipients remains understudied.

**Methods.:**

This retrospective cohort study used TriNetX data (January 2010–December 2023) to compare outcomes in liver transplant recipients prescribed GLP1RAs (semaglutide, dulaglutide, liraglutide) within 1-mo posttransplant (n = 546) versus nonusers (n = 37 153). Propensity score matching (1:1) balanced demographics, comorbidities, and medications (n = 541 per group). Outcomes included mortality, hospitalizations, cardiovascular/renal/respiratory events, and graft outcomes.

**Results.:**

Over a mean follow-up 838.5 d (SD 291.9) in the GLP1RA cohort and 884.3 d (SD 313.7) in the non-GLP1RA group, GLP1RA use was associated with a 43% lower all-cause mortality (7.0% versus 12.9%; hazard ratio [HR], 0.566; 95% confidence interval [CI], 0.381-0.841) and 39% fewer hospitalizations (60.4% versus 74.5%; HR, 0.613; 95% CI, 0.530-0.710). Acute heart failure (HR, 0.386; 95% CI, 0.285-0.524), renal failure/dialysis (HR, 0.489; 95% CI, 0.413-0.579), and respiratory failure (HR, 0.484; 95% CI, 354-0.662) risks were significantly reduced. No differences were observed in graft failure/rejection, myocardial infarction, stroke, atrial fibrillation/flutter, ventricular tachycardia, or ischemic optic neuropathy.

**Conclusions.:**

Early GLP1RA initiation in liver transplant recipients was associated with reduced mortality, hospitalizations, respiratory, and cardiorenal complications without compromising graft safety. These findings support GLP1RAs as a promising adjunct therapy, warranting prospective trials to confirm benefits in this high-risk population.

## INTRODUCTION

Liver transplantation remains the definitive and often life-saving treatment for patients with end-stage liver disease; however, long-term survival in this population is complicated by increased incidence of nonhepatic complications.^1^ After transplantation, recipients frequently face an elevated risk of cardiometabolic disease, driven by the combined effects of weight gain, insulin resistance, and hypertension.^2^ In addition, the use of immunosuppressive therapy such as calcineurin inhibitors, although essential for maintaining graft integrity, exert nephrotoxic effects as well as direct adverse effects on glucose metabolism and lipid regulation, thereby accelerating the development of adverse cardiovascular outcomes in an already high-risk cardiovascular population.^3-5^ Furthermore, obesity and diabetes often develop or significantly worsen after transplantation, creating a self-reinforcing cycle of metabolic dysfunction that threatens both graft longevity and overall patient survival.^2^

Glucagon-like peptide-1 receptor agonists (GLP1RAs), initially developed for glycemic control, have emerged as effective therapies for metabolic and cardiovascular disease.^2^ Beyond stimulating insulin secretion and suppressing glucagon, these agents promote weight loss through delayed gastric emptying and appetite suppression, enhance myocardial glucose utilization, reduce oxidative stress, and mitigate maladaptive cardiac remodeling.^6^ They also improve endothelial function by stimulating nitric oxide release and modulating blood pressure through natriuresis and vasodilation.^7^

Despite these advances, the role of GLP1RAs in liver transplant recipients remains poorly studied. While newer studies suggest that GLP1RAs are well tolerated in transplant recipients and result in weight loss and better glycemic control, cardiovascular and renal outcomes have not been systematically evaluated in this population.^8^

In this study, we evaluated the effect of GLP1RA therapy on all-cause mortality, all-cause hospitalizations, cardiovascular events, renal dysfunction, and graft function in patients after liver transplantation.

## MATERIALS AND METHODS

### Study Design and Population

This retrospective cohort study utilized de-identified electronic health records (EHRs) from the TriNetX Research Network, a global database spanning >100 healthcare organizations. Adults aged ≥18 years who underwent liver transplantation between January 1, 2010, and December 31, 2023, were included. Patients were required to be on guideline-directed dual immunosuppressive therapy, defined as concurrent use of a calcineurin inhibitor (tacrolimus or cyclosporine) and an antiproliferative agent (mycophenolate, azathioprine, or sirolimus).^9^

The exposed cohort comprised patients who initiated GLP1RA therapy (semaglutide, dulaglutide, or liraglutide) within 1 mo posttransplant to minimize immortal time bias. The unexposed cohort included patients without any recorded GLP1RA use during the study period. Individuals with prior GLP1RA use before transplantation were excluded. A look-back period was also completed to ensure all patients had appropriate documentation of baseline characteristics, and those with no documented healthcare visits were excluded.

### Baseline Characteristics and Risk Factors

Demographic variables, comorbidities, laboratory values, and medication use were extracted from the EHRs. Demographic variables included age, sex, race/ethnicity (White, Black or African American, Hispanic or Latino), and socioeconomic status. Comorbidities encompassed diabetes mellitus, hypertensive diseases, disorders of lipoprotein metabolism, obesity, chronic kidney disease (CKD), ischemic heart diseases, nicotine dependence, sleep apnea, heart failure (HF), alcohol-related disorders, atrial fibrillation/flutter, and prior cerebral infarction.

Pharmacotherapy use included insulin, metformin, glipizides, antiarrhythmics, anticoagulants, beta-blockers, loop diuretics, platelet aggregation inhibitors, antilipemic agents, calcium channel blockers, spironolactone, thiazide diuretics, angiotensin-converting enzyme inhibitors, angiotensin II receptor blockers, angiotensin receptor-neprilysin inhibitors, and sodium-glucose cotransporter-2 inhibitors.

Laboratory and clinical variables comprised serum creatinine, body mass index (BMI), low-density lipoprotein (LDL) cholesterol, hemoglobin A1c (HbA1c), N-terminal pro-B-type natriuretic peptide, and left ventricular ejection fraction. We also adjusted for Z-coding, which is an International Classification of Diseases, Tenth Revision (ICD-10) coded system (Z55-65) to account for social hazards to health.

### Clinical Outcomes

The event index date was 1 mo after liver transplantation. The primary study outcomes included all-cause mortality and all-cause hospitalizations, as well as secondary clinical outcomes including acute HF events (ICD-10 I50.21, I50.23, I50.31, I50.33, I50.41, or I50.43), stroke (ICD-10 I63), acute myocardial infarction (MI) (I21.0-I21.4, I21.9), cardiac arrest (ICD-10 I46), new atrial fibrillation/flutter (AF) (ICD-10 I48), ventricular tachycardia (ICD-10 I47.2), acute renal failure or the need for dialysis (ICD-10 N17, N18.6, CPT codes: 90945, 1012752, 90947, 90940), liver transplant failure (ICD-10: T86.42), liver transplant rejection (ICD-10: T86.41), and respiratory failure (ICD-10: J96). We also included ischemic optic neuropathy (ICD-10: H46) as an outcome given recent observational data suggesting nonarteritic anterior optic neuropathy as an adverse event in patients on GLP1RA therapy.^10^ We also included follow-up LDL, HbA1C, and BMI. Follow-up was censored at the occurrence of an event, death, or the last recorded encounter within the study period. Groups were compared according to GLP1RA use.

### Statistical Analysis

Nominal variables are presented as frequencies and percentages. Continuous variables are reported as mean ± SD for normally distributed data or median (interquartile range) for nonnormally distributed data. Baseline characteristics were compared between the GLP1RA and non-GLP1RA cohorts using chi-square tests for categorical variables and independent-sample *t*-tests or Mann-Whitney U tests for continuous variables, as appropriate.

A 1:1 propensity score matching (PSM) was performed using a greedy nearest-neighbor algorithm with a caliper width of 0.1. The PSM model included baseline demographics, comorbidities, and baseline medications. Time-to-event outcomes were analyzed using Kaplan-Meier survival curves with log-rank testing. Cox proportional hazards regression models were used to calculate hazard ratios (HRs) and 95% confidence intervals (CIs). A two-tailed *P* value <0.05 was deemed statistically significant. All analyses were conducted using R software (v4.3.2, R Foundation for Statistical Computing, Vienna, Austria) within the TriNetX platform. The Mayo Clinic Institutional Review Board deemed this study exempt from approval given the nature of the publicly available and anonymized data (Institutional Review Board #25-008530). Accordingly, individual participant consent was not required.

## RESULTS

Before PSM, 546 liver transplant recipients prescribed GLP1RAs within 1 mo posttransplant were compared with 37 153 non-GLP1RA users. The GLP1RA cohort was older (mean age 62.9 versus 60.1 y, *P* < 0.001) and had a higher prevalence of diabetes mellitus (92.1% versus 37.2%, *P* < 0.001), hypertensive diseases (89.9% versus 57.8%, *P* < 0.001), obesity (70.0% versus 23.4%, *P* < 0.001), and CKD (62.5% versus 39.5%, *P* < 0.001). The GLP1RA group also included higher baseline use of insulin (93.2% versus 59.1%, *P* < 0.001), metformin (45.8% versus 7.6%, *P* < 0.001), and cardiovascular therapies such as beta-blockers (84.8% versus 61.0%, *P* < 0.001) and anticoagulants (85.2% versus 64.9%, *P* < 0.001). However, over the follow-up period, only 18% and 22% of the GLP1RA and non-GLP1RA cohorts were on anticoagulation, respectively. Laboratory and clinical parameters revealed higher HbA1c (7.0% versus 5.7%, *P* < 0.001), BMI (33.2 versus 28.2 kg/m^2^, *P* < 0.001), and lower serum creatinine (1.4 versus 1.6 mg/dL, *P* = 0.010) in the GLP1RA cohort. After 1:1 PSM, 541 patients were included in each group, with well-balanced baseline characteristics. Baseline characteristics of the study cohort are reported in Table 1.

**TABLE 1.** Baseline Characteristics

	Pre-PSM			Post-PSM		
	On GLP1RAs, n (%)	Not on GLP1RAs, n (%)	*P*	On GLP1RAs, n (%)	Not on GLP1RAs, n (%)	*P*
Demographics						
Current age, y (mean ± SD)	62.9 ± 11.2	60.1 ± 16.3	<0.001	62.9 ± 11.3	63.0 ± 12.0	0.990
Female	230 (42.1)	13 793 (37.1)	0.020	228 (42.1)	238 (44.0)	0.540
White	385 (70.5)	25 299 (68.1)	0.230	380 (70.2)	382 (70.6)	0.890
Hispanic or Latino	116 (21.2)	4407 (11.9)	<0.001	115 (21.3)	98 (18.1)	0.190
Black or African American	57 (10.4)	4353 (11.7)	0.360	57 (10.5)	62 (11.5)	0.630
Comorbidities						
Diabetes mellitus	503 (92.1)	13 814 (37.2)	<0.001	498 (92.0)	495 (91.5)	0.740
Hypertensive diseases	491 (89.9)	21 473 (57.8)	<0.001	486 (89.8)	476 (88.0)	0.330
Disorders of lipoprotein metabolism	406 (74.4)	11 286 (30.4)	<0.001	401 (74.1)	398 (73.6)	0.840
Overweight and obesity	382 (70.0)	8711 (23.4)	<0.001	377 (69.7)	391 (72.3)	0.350
Chronic kidney disease	341 (62.5)	14 658 (39.5)	<0.001	338 (62.5)	333 (61.5)	0.750
Ischemic heart diseases	269 (49.3)	9074 (24.4)	<0.001	267 (49.4)	272 (50.3)	0.760
Nicotine dependence	192 (35.2)	7790 (21.0)	<0.001	191 (35.3)	196 (36.2)	0.750
Sleep apnea	183 (33.5)	4040 (10.9)	<0.001	179 (33.1)	187 (34.6)	0.610
Heart failure	142 (26.0)	4575 (12.3)	<0.001	139 (25.7)	152 (28.1)	0.370
Alcohol-related disorders	132 (24.2)	8046 (21.7)	0.160	130 (24.0)	140 (25.9)	0.480
Atrial fibrillation and flutter	85 (15.6)	3785 (10.2)	<0.001	85 (15.7)	87 (16.1)	0.870
Cerebral infarction	47 (8.6)	1084 (2.9)	<0.001	45 (8.3)	46 (8.5)	0.910
Persons with potential health hazards related to socioeconomic circumstances (Z-Codes)	45 (8.2)	1282 (3.5)	<0.001	44 (8.1)	44 (8.1)	1.000
Pharmacotherapies						
Insulin	509 (93.2)	21 937 (59.1)	<0.001	504 (93.2)	511 (94.5)	0.380
Metformin	250 (45.8)	2824 (7.6)	<0.001	245 (45.3)	177 (32.7)	<0.001
Glipizides	92 (16.9)	1400 (3.8)	<0.001	89 (16.4)	91 (16.8)	0.870
Antiarrhythmics	473 (86.6)	24 706 (66.5)	<0.001	468 (86.5)	470 (86.9)	0.860
Anticoagulants	465 (85.2)	24 115 (64.9)	<0.001	460 (85.0)	466 (86.1)	0.600
Beta-blockers	463 (84.8)	22 664 (61.0)	<0.001	459 (84.8)	464 (85.8)	0.670
Loop diuretics	443 (81.1)	23 952 (64.5)	<0.001	438 (81.0)	442 (81.7)	0.750
Platelet aggregation inhibitors	401 (73.4)	13 535 (36.4)	<0.001	396 (73.2)	392 (72.5)	0.780
Antilipemic agents	391 (71.6)	10 526 (28.3)	<0.001	386 (71.3)	391 (72.3)	0.740
Calcium channel blockers	374 (68.5)	13 437 (36.2)	<0.001	369 (68.2)	350 (64.7)	0.220
Spironolactone	280 (51.3)	14 301 (38.5)	<0.001	275 (50.8)	291 (53.8)	0.330
Thiazides	181 (33.1)	6114 (16.5)	<0.001	179 (33.1)	183 (33.8)	0.800
ACE inhibitors	205 (37.5)	5856 (15.8)	<0.001	200 (37.0)	198 (36.6)	0.900
Angiotensin II blockers	165 (30.2)	3929 (10.6)	<0.001	163 (30.1)	176 (32.5)	0.390
ARNI	10 (1.8)	101 (0.3)	<0.001	10 (1.9)	10 (1.9%)	1.000
Sodium-glucose cotransporter 2 inhibitors	120 (22.0)	572 (1.5)	<0.001	115 (21.3)	109 (20.1)	0.650
Labs and clinical variables						
Creatinine in serum (mean ± SD)	1.4 ± 1.0	1.6 ± 1.6	0.010	1.4 ± 1.0	1.6 ± 1.3	<0.001
BMI (mean ± SD)	33.2 ± 6.2	28.2 ± 6.4	<0.001	33.2 ± 6.2	31.5 ± 6.3	<0.001
Cholesterol in LDL (mean ± SD)	84.4 ± 40.7	83.6 ± 56.0	0.760	84.2 ± 40.3	78.9 ± 42.6	0.060
Hemoglobin A1c (mean ± SD)	7.0 ± 1.8	5.7 ± 1.5	<0.001	7.0 ± 1.8	6.5 ± 1.6	<0.001
Natriuretic peptide.B prohormone N-terminal (mean ± SD)	1529.9 ± 3205.1	3607.6 ± 8713.1	0.040	1412.3 ± 3058.2	2989.5 ± 5732.6	0.040
Left ventricular ejection fraction (%) (mean ± SD)	60.0 ± 10.3	61.8 ± 9.2	0.210	60.0 ± 10.3	62.0 ± 8.0	0.300

Continuous variables are reported as mean and standard deviation for normally distributed data or median and interquartile range for nonnormally distributed data; categorical data are reported at frequency and percentage.

ACE, angiotensin-converting enzyme; ASCVD, atherosclerotic cardiovascular disease; CAD, coronary artery disease; GLP1RA, glucagon-like peptide-1 receptor agonist; HDL-C, high-density lipoprotein cholesterol; LDL-C, low-density lipoprotein cholesterol; Lp(a), lipoprotein(a) ; PAD, peripheral arterial disease; PSM, propensity score matching; T2DM, type 2 diabetes mellitus; TIA, transient ischemic attack.

At follow-up, LDL levels were lower in the GLP-1RA cohort compared with the non-GLP-1RA cohort (75.62 ± 35.59 mg/dL versus 83.09 ± 37.75 mg/dL; *t* = –2.83; *P* < 0.004). BMI was also lower among patients receiving GLP-1RA therapy (28.12 ± 6.30 kg/m^2^ versus 30.80 ± 6.29 kg/m^2^; *t* = 3.144; *P* = 0.002). HbA1c levels were similar between groups, with no statistically significant difference (6.85% ± 1.63% versus 6.84% ± 1.65%; *t* = 0.104; *P* = 0.918).

**FIGURE 1.**
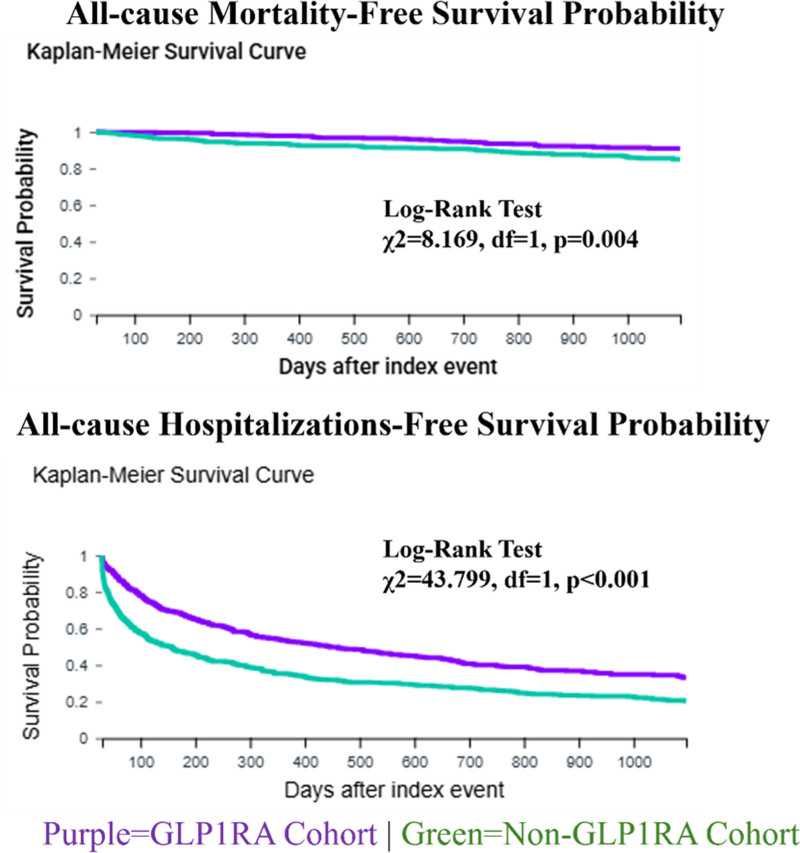
Kaplan-Meier curve. Event-free survival analyses for all-cause mortality and hospitalization outcomes.

Over a mean follow-up of 838.5 d (SD 291.9) in the GLP1RA cohort and 884.3 d (SD 313.7) in the non-GLP1RA cohort, patients treated with GLP1RAs demonstrated significantly lower risks of all-cause mortality and hospitalization (Table 2). The endpoint of all-cause mortality occurred in 38 (7.0%) patients in the GLP1RA group compared with 70 (12.9%) patients in the non-GLP1RA group, corresponding to a 43% risk reduction (HR, 0.566; 95% CI, 0.381-0.841). Similarly, all-cause hospitalizations occurred in 327 patients in the GLP1RA group compared with 403 patients in the non-GLP1RA group (60.4% versus 74.5%; HR, 0.613; 95% CI, 0.530-0.710) (Figure 1).

**TABLE 2.** Outcomes

Outcome	With GLP1RAs events, n (%)	Without GLP1RAs events, n (%)	Hazard ratio (95% CI)	Event-free survival probabilities (GLP1RA vs without)
All-cause mortality	38 (7.0)	70 (12.9)	0.566 (0.381, 0.841)	90.8% vs 85.1%
All-cause hospitalizations	327 (60.4)	403 (74.5)	0.613 (0.530, 0.710)	33.1% vs 20.3%
Acute heart failure events	59 (10.9)	142 (26.2)	0.386 (0.285, 0.524)	87.2% vs 71.0%
Acute myocardial infarction	23 (4.3)	35 (6.5)	0.671 (0.397, 1.137)	94.9% vs 92.4%
Stroke	26 (4.8)	39 (7.2)	0.675 (0.411, 1.110)	94.2% vs 91.9%
Cardiac arrest	<10 (1.8)	13 (2.4)	0.626 (0.259, 1.511)	98.1% vs 97.4%
Ventricular tachycardia	<10 (1.8)	11 (2.0)	0.942 (0.400-2.220)	97.8% vs 97.6%
New atrial fibrillation/flutter	83 (15.3)	92 (17.0)	0.907 (0.674, 1.220)	83.0% vs 81.4%
Acute renal failure or need for dialysis	223 (41.2)	346 (64.0)	0.489 (0.413, 0.579)	54.9% vs 31.4%
Liver transplant failure	24 (4.4)	37 (6.8)	0.651 (0.389, 1.089)	94.5% vs 92.3%
Liver transplant rejection	80 (14.8)	77 (14.2)	1.055 (0.772, 1.443)	83.0% vs 84.1%
Ischemic optic neuropathy	<10 (1.8)	<10 (1.8)	0.986 (0.062, 15.760)	99.8% vs 99.8%
Respiratory failure	59 (10.9)	116 (21.4)	0.484 (0.354, 0.662)	87.1% vs 75.9%
Follow-up labs				
Outcomes	GLP1RA cohort	Non-GLP1RA cohort	T statistics	*P*
LDL	75.62 (SD 35.59)	83.09 (SD 37.75)	−2.83	<0.004
BMI	28.12 (SD 6.30)	30.80 (SD 6.29)	3.144	0.002
HbA1C	6.85 (SD 1.63)	6.84 (SD 1.65)	0.104	0.918

BMI, body mass index; GLP1RA, glucagon-like peptide-1 receptor agonist; HbA1c, hemoglobin A1c; LDL, low-density lipoprotein.

**FIGURE 2.**
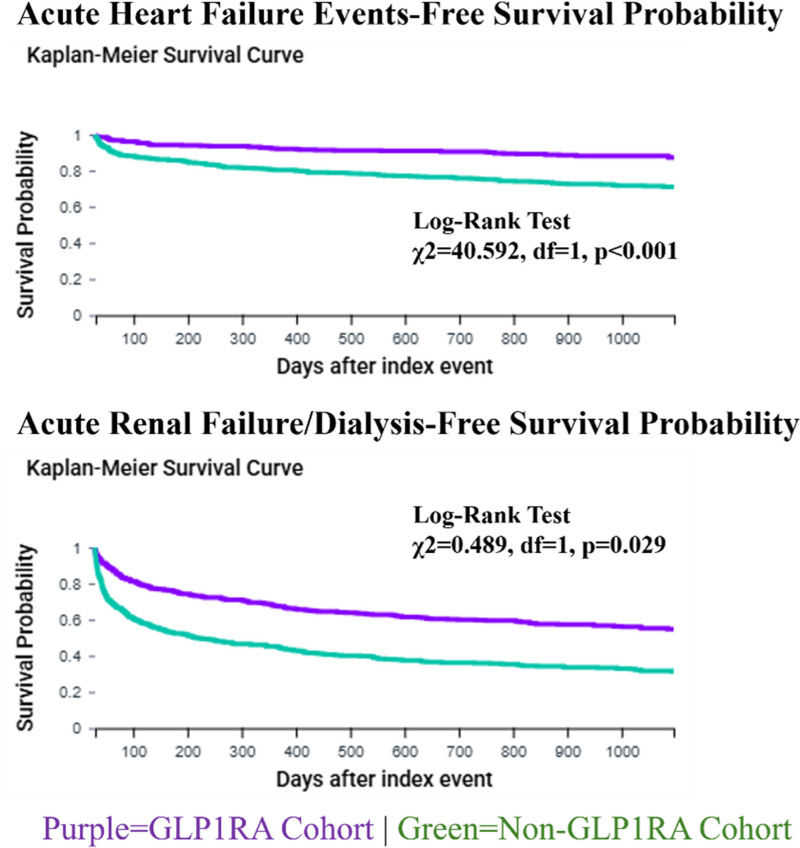
Kaplan-Meier curve. Event-free survival analyses for acute heart failure events and acute renal failure/dialysis outcomes.

GLP1RA use was associated with a 61% lower risk of acute HF (59 [10.9%] versus 142 [26.2%]; HR, 0.386; 95% CI, 0.285-0.524) and a 51% reduction in acute renal failure or dialysis requirement (223 [41.2%] versus 346 [64.0%]; HR, 0.489; 95% CI, 0.413-0.579) (Figure 2). Respiratory failure events were also significantly lower in the GLP1RA group (59 [10.9%] versus 116 [21.4%]; HR, 0.484; 95% CI, 0.354-0.662).

**FIGURE 3.**
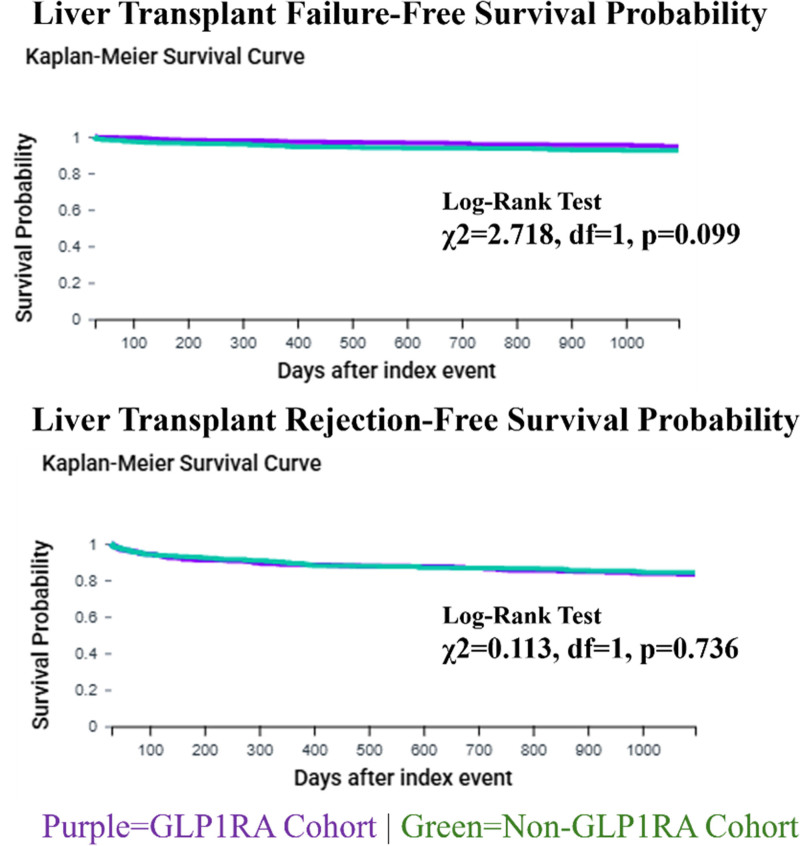
Kaplan-Meier curve. Event-free survival analyses for liver transplant failure and rejection outcomes.

No significant differences were observed in acute MI (23 [4.3%] versus 35 [6.5%]; HR, 0.671; 95% CI, 0.397-1.137), stroke (26 [4.8%] versus 39 [7.2%]; HR, 0.675; 95% CI, 0.411-1.110), cardiac arrest (<10 [1.8%] versus 13 [2.4%]; HR, 0.626; 95% CI, 0.259-1.511), or ventricular tachycardia (<10 [1.8%] versus 11 [2.0%]; HR, 0.942; 95% CI, 0.400-2.220). Liver transplant failure (24 [4.4%] versus 37 [6.8%]; HR, 0.651; 95% CI, 0.389-1.089) and rejection rates (80 [14.8%] versus 77 [14.2%]; HR, 1.055; 95% CI, 0.772-1.443) also did not differ between groups (Figure 3). Similarly, the risk of ischemic optic neuropathy was insignificant between the 2 cohorts (HR, 0.986; 95% CI, 0.062-15.760).

## DISCUSSION

In our cohort of liver transplant recipients, the initiation of GLP1RA therapy was associated with significantly improved outcomes. Patients receiving GLP1RA had lower all-cause mortality and fewer all-cause hospitalizations compared with nonusers. Moreover, the GLP1RA group experienced fewer acute HF events and acute renal failure/dialysis events. These findings align with emerging data in other transplant populations.^11,12^

Our study showed similar mortality benefits seen in other GLP1RA studies involving transplant patients. For example, Dotan et al reported a significant mortality reduction with GLP1RA in a mixed transplant cohort,^11^ and Lin et al similarly found a significant mortality benefit in kidney transplant recipients on GLP1RA.^12^ The reduced hospitalization rate in our study likely stems from fewer acute events (HF, renal failure) and perhaps better overall metabolic health status. GLP1RA-treated patients in our study also had significantly lower risk of acute HF events. A recent meta-analysis of HF with preserved ejection fraction trials found that GLP1RAs significantly reduced the composite of cardiovascular death or HF hospitalization and lowered worsening HF events.^13^ This may be explained by GLP1RAs’ ability to improve multiple cardiovascular risk factors that contribute to HF since they promote significant weight loss, improved blood pressure and lipid profiles.^7^ GLP1RAs also improve endothelial function and reduce oxidative stress and inflammation, contributing to their antiatherogenic effects.^7^ In the myocardium, GLP1RAs can enhance myocardial glucose utilization and reduce maladaptive remodeling.^6^

Moreover, the significant decrease in risk of acute renal failure or need for dialysis is consistent with known renoprotective effects of GLP1RA. A recent meta-analysis showed that GLP1RAs reduce the risk of a composite kidney outcome consisting of kidney failure (kidney replacement therapy or a persistent estimated glomerular filtration rate (eGFR) <15 mL/min per 1·73 m^2^, a sustained reduction in eGFR by at least 50% or the nearest equivalent, or death from kidney failure,^14^ likely through improving glycemic control, reducing hyperfiltration, and attenuating inflammation.^15^ In addition, GLP1RAs reduce albuminuria and slow decline in eGFR in diabetic kidney disease.^15^ In the context of calcineurin inhibitor nephrotoxicity, these benefits may be especially valuable.

The lack of differences in graft failure or rejection may suggest that adding GLP1RA does not adversely affect the immune function or graft viability. Similarly, studies that evaluated GLP1RA use in patients with known liver transplantation tolerated immunosuppresive therapies well without necessitating changes in immunosuppressive therapy.^16,17^ For instance, Grancini et al found no need to adjust calcineurin inhibitor dosing when adding GLP1RA, and adverse events were minimal.^16^ Similarly, heart transplant recipients on GLP1RA rarely required any immunosuppression dose changes.^17^ There is also no evidence of direct immunostimulation by GLP-1Ras. In fact, no immune-related adverse events or rejection signals have emerged in the available posttransplant clinical data.^18^ Preclinical findings even suggest GLP-1R signaling may have a tolerogenic effect on T cells, helping to mitigate alloimmune responses.^19^ Beyond their glycemic effects, GLP-1RAs may indirectly improve graft health by alleviating metabolic stress, fatty infiltration, and inflammation.^16^ Importantly, liver stiffness (a marker of fibrosis) decreased with GLP1-RA therapy, suggesting less graft steatosis/fibrosis with GLP-1RA treatment.^16^ Thus, GLP1RAs appear safe in transplant patients, without evidence of immunologic interactions.

Our study provides evidence that GLP1RA therapy may offer multifactorial benefits in liver transplant recipients, potentially improving survival and promoting cardiorenal stability without compromising graft safety. By mimicking endogenous GLP-1, these drugs increase glucose-dependent insulin secretion and suppress glucagon, improving glycemic control, among multiple other beneficial effects.^6^ This positions GLP1RAs as a promising adjunctive therapy in post-liver transplant patients. However, given the observational nature of our study, these findings require cautious interpretation and warrant further investigation through prospective, controlled trials.

Several limitations in our study exist. Our study is retrospective and observational which precludes causal inference. Furthermore, residual confounding cannot be excluded despite propensity matching. Moreover, the TriNetX database lacks granular patient data, and there is a potential of misclassification or under-coding of data with ICD diagnosis coding. To that point, we are unable to identify specific causes of death or the exact indications for liver transplantation and causes of the end-stage liver disease, although the majority of our patients had high burden of metabolic comorbidities. Acuity of the perioperative state during liver transplantation, blood type, and underlying severity of systemic illness are not available. In addition, our cohort comprised liver transplant recipients with a substantial burden of cardiometabolic comorbidities, including diabetes, obesity, hypertension, and CKD, which may lead to selection bias. As such, the findings may reflect outcomes in metabolically high-risk recipients and may not be generalizable to the broader liver transplant population. We also lacked data on medication adherence and do not have data on longitudinal medication prescriptions, as it cannot be verified in EHR databases through the TriNetX network. Similarly, we do not have the initial indication for GLP1RA use; although the majority of the GLP1RA cohort had a diabetic range mean HbA1C and a BMI >30 kg/m^2^. Finally, follow-up time was limited to approximately three years given skewed follow-up data among the 2 cohorts if extended any further.

## CONCLUSIONS

Our findings suggest that GLP1RA therapy after liver transplantation is associated with better long-term outcomes, particularly in reducing mortality, HF, hospitalizations, renal failure, and respiratory failure events. Prospective studies and randomized trials are needed to confirm these benefits and to assess long-term cardiovascular and renal outcomes in transplant recipients.
